# Editorial: Computational Epitranscriptomics: Bioinformatic Approaches for the Analysis of RNA Modifications

**DOI:** 10.3389/fgene.2020.630360

**Published:** 2020-12-11

**Authors:** Erik Dassi, Pavel V. Baranov, Mattia Pelizzola

**Affiliations:** ^1^Laboratory of RNA Regulatory Networks, Department of Cellular, Computational and Integrative Biology (CIBIO), University of Trento, Trento, Italy; ^2^School of Biochemistry and Cell Biology, University College Cork, Cork, Ireland; ^3^Shemyakin-Ovchinnikov Institute of Bioorganic Chemistry, Russian Academy of Science, Moscow, Russia; ^4^Center for Genomic Science, Fondazione Istituto Italiano di Tecnologia, Milan, Italy

**Keywords:** RNA modification, bioinformatics and computational biology, RNA methylation, RNA editing, pseudouridine (Ψ), nanopore

RNA modifications were discovered decades ago, and more than 150 different marks have been found decorating various RNA species, including coding and non-coding transcripts (Boccaletto et al., 2018). Yet, only in the last decade this research field rapidly expanded, due to the development of simple and effective methods for the genome-wide identification of some of these marks, such as MeRIP-seq for the profiling of N6-methyladenosine (m6A) (Dominissini et al., 2012; Meyer et al., 2012). The renowned interest in the field led to the identification of key effectors—writers, erasers, and readers—that establish and decode the patterning of specific marks. This suggested that RNA modifications have the potential to be dynamically controlled, similarly to their genomic counterparts, the modifications of DNA and chromatin. Indeed, in analogy to the epigenome, the collective set of RNA modifications was named epitranscriptome. Altogether, the epitranscriptome is considered an important determinant of RNA fate, and specific marks were found to be involved in various steps of the RNA life cycle including, while not limited to, transcription, processing, decay, and translation (Roundtree et al., 2017).

As often occurs, following the birth of a new omics, the development of computational methods that are tailored to the analysis of those high-throughput datasets started to flourish. In analogy to the development of computational epigenomics (Bock and Lengauer, 2008), this research field could be referred to as computational epitranscriptomics.

This Research Topic collects a number of contributions in this field. Few manuscripts focused on the development of novel methods for the prediction of RNA modifications. A Galaxy-based user friendly graphical workflow was developed that cover the preprocessing of omics data, the quantification of mismatch and arrest rates with single-nucleotide resolution, and the subsequent machine learning, modification calling and visualization (Schmidt et al.). A computational workflow dedicated to 2′-O-methylation marks was optimized, allowing a more accurate detection of these marks and a more precise quantification of their level variations (Pichot et al.). A novel tool (LITOPHONE) was developed that adopts an ensemble predictor relying on sequence features to predict m6A sites in long non-coding RNAs (Liu L. et al.). A web server (PIANO) was implemented that relies on various genomic features, including sequence information, for the prediction and functional annotation of pseudouridine sites (Song et al.). Finally, a bioinformatic pipeline (tRFs-Galaxy) was developed for the study of small non-coding RNAs derived from tRNAs (tRFs), allowing the study of tRFs biogenesis in *Drosophila melanogaster* (Molla-Herman et al.).

Two additional contributions discussed pitfalls in the analysis of specific marks. A first study discussed the impact of different bioinformatics steps on the detection of RNA editing events, describing key metrics for the quantification of their level of activity (Giudice et al.). A second contribution compared m6A genome-wide maps generated in various studies based on eight different methods, discussing the agreement of the data and the challenges in their comparative analysis, revealing an expression bias in the detected genes (Capitanchik et al.).

Two contributions were focused on the use of direct RNA sequencing through the Nanopore platform that enables long-reads sequencing of native transcripts. A perspective discussed how these data could allow quantifying the dynamics of modified RNAs at the level of individual isoforms (Furlan et al.). A second study introduced MasterOfPores, a NextFlow workflow that facilitates the analysis of these data, allowing the prediction of RNA modifications and the estimation of polyA tail lengths (Cozzuto et al.).

Finally, three different studies introduced bioinformatics workflows for studying the impact of RNA modifications in various tumor types. In the first study, a workflow based on consensus clustering and gene set enrichment analysis was presented that allowed the subsequent construction of a prognostic risk model suggesting the involvement of three m6A-related genes in liver cancer (Wang et al.). In the second study, bioinformatics analyses revealed a risk signature based on three m6A regulators, proposing candidate prognostic markers predictor of the clinicopathological features in hepatocellular carcinoma (Liu W. et al.). In the third study, integrated bioinformatics analyses led to the identification of differentially expressed transcripts with aberrant methylation patterns in malignant pheochromocytoma (Lin et al.).

Despite the rapid advance of the field, which allowed expanding the set of known marks, profiling their pattern, and disclosing their functional roles, a number of open questions remain (Frye et al., 2016). Most modifications remain poorly characterized, it is unclear whether different marks crosstalk and whether an epitranscriptional code exists. We are only starting to understand how, where and when these modifications are altered and whether they represent potential therapeutic targets in diseases. Key for answering these and other questions will be the continuous development of methods to map and analyze these marks. This research would benefit from the establishment of large scale collaborative and networking efforts such as the European Epitranscriptomics Network (www.epitran.eu) (Jantsch et al., 2018).

## Author Contributions

All authors contributed writing this Editorial and managing the corresponding Research Topic.

## Conflict of Interest

The authors declare that the research was conducted in the absence of any commercial or financial relationships that could be construed as a potential conflict of interest.

## References

[B1] Boccaletto P. Machnicka M. A. Purta E. Piatkowski P. Baginski B. Wirecki T. K.. (2018). MODOMICS: a database of RNA modification pathways. 2017 update. Nucleic Acids Res. 46, D303–D307. 10.1093/nar/gkx1030 29106616 PMC5753262

[B2] Bock C. Lengauer T. (2008). Computational epigenetics. Bioinformatics 24, 1–10. 10.1093/bioinformatics/btm546 18024971

[B3] Dominissini D. Moshitch-Moshkovitz S. Schwartz S. Salmon-Divon M. Ungar L. Osenberg S.. (2012). Topology of the human and mouse m6A RNA methylomes revealed by m6A-seq. Nature 485, 201–206. 10.1038/nature11112 22575960

[B4] Frye M. Jaffrey S. R. Pan T. Rechavi G. Suzuki T. (2016). RNA modifications: what have we learned and where are we headed? Nat. Rev. Genet. 17, 365–372. 10.1038/nrg.2016.47 27140282

[B5] Jantsch M. F. Quattrone A. O'Connell M. Helm M. Frye M. Macias-Gonzales M.. (2018). Positioning Europe for the EPITRANSCRIPTOMICS challenge. RNA Biol. 15, 829–831. 10.1080/15476286.2018.1460996 29671387 PMC6152430

[B6] Meyer K. D. Saletore Y. Zumbo P. Elemento O. Mason C. E. Jaffrey S. R. (2012). Comprehensive analysis of mRNA methylation reveals enrichment in 3' UTRs and near stop codons. Cell 149, 1635–1646. 10.1016/j.cell.2012.05.003 22608085 PMC3383396

[B7] Roundtree I. A. Evans M. E. Pan T. He C. (2017). Dynamic RNA modifications in gene expression regulation. Cell 169, 1187–1200. 10.1016/j.cell.2017.05.045 28622506 PMC5657247

